# A Revised Point-to-Point Calibration Approach with Adaptive Errors Correction to Weaken Initial Sensitivity of Cuff-Less Blood Pressure Estimation

**DOI:** 10.3390/s20082205

**Published:** 2020-04-13

**Authors:** Jiang Shao, Ping Shi, Sijung Hu, Hongliu Yu

**Affiliations:** 1 Institute of Rehabilitation Engineering and Technology, University of Shanghai for Science and Technology, Shanghai 200093, China; 2 Wolfson School of Mechanical, Electrical and Manufacturing Engineering, Loughborough University, Loughborough, Leicestershire LE11 3TU, UK

**Keywords:** point-to-point pairing method, initial sensitivity, penalty factor, correcting errors, cuff-less blood pressure

## Abstract

Initial calibration is a great challenge for cuff-less blood pressure (BP) measurement. The traditional one point-to-point (oPTP) calibration procedure only uses one sample/point to obtain unknown parameters of a specific model in a calm state. In fact, parameters such as pulse transit time (PTT) and BP still have slight fluctuations at rest for each subject. The conventional oPTP method had a strong sensitivity in the selection of initial value. Yet, the initial sensitivity of calibration has not been reported and investigated in cuff-less BP motoring. In this study, a mean point-to-point (mPTP) paring calibration method through averaging and balancing calm or peaceful states was proposed for the first time. Thus, based on mPTP, a factor point-to-point (fPTP) paring calibration method through introducing the penalty factor was further proposed to improve and optimize the performance of BP estimation. Using the oPTP, mPTP, and fPTP methods, a total of more than 100,000 heartbeat samples from 21 healthy subjects were tested and validated in the PTT-based BP monitoring technologies. The results showed that the mPTP and fPTP methods significantly improved the performance of estimating BP compared to the conventional oPTP method. Moreover, the mPTP and fPTP methods could be widely popularized and applied, especially the fPTP method, on estimating cuff-less diastolic blood pressure (DBP). To this extent, the fPTP method weakens the initial calibration sensitivity of cuff-less BP estimation and fills in the ambiguity for individualized calibration procedure.

## 1. Introduction

Uncontrolled hypertension or high blood pressure (BP) was a major risk factor that links to the potential development of serious diseases such as stroke, hypertensive heart disease, and coronary artery disease [1]. BP was influenced by many factors such as food, exercise, mental situations, and stress, among others; thus, it varied considerably from time to time [2]. Instantaneous information about BP status could be obtained from conventional standard cuff-based BP measurement, such as oscillometry [3] and auscultation. However, these methods were not applicable to ambulatory BP monitoring (ABPM) or home BP monitoring (HBPM) due to the cumbersome and discontinuous nature of cuff wrapping around the arm or leg to detect the oscillations during cuff-deflation [4] and the limited frequency of measurement [5]. Moreover, whether the clinical BP reflects the normal level due to the white coat effect had been questioned [6]. Therefore, the development of cuff-less BP monitoring technology was extremely urgent.

For decades, many directions, such as biosensor [7,8], signal processing [9], calibrating, or modeling [10] and performance verification [11] were extensively reported and investigated for cuff-less BP measurement. Various BP monitoring models were proposed based on pulse wave velocity (PWV), pulse transit time (PTT), and pulse wave analysis (PWA), in which pulse signals could be non-invasively obtained through electrocardiogram (ECG), non-invasive photoplethysmography (PPG) [3,8] and ballistocardiography (BCG) originated from a cardiovascular system. Here, PPG, a non-invasive optical measurement technique by means of photoelectric measurement, obtained physiological signals and characteristics of the human body by detecting changes in blood volume in microvessels. The PTT was the time delay for the pressure wave to travel between two arterial sites and could be estimated by an R peak time interval between ECG and PPG signal in same cardiac cycle [7,8,9]. The PTT-based cuff-less BP estimation measurement had shown great potential and attracted extensive attention [12,13,14], especially while estimating systolic blood pressure (SBP) [12,15]. For example, early research by Chen et al. [16] had developed a PTT-BP model based on the Moens–Korteweg (M-K) equation [9,10,12,13,14], and showed that PTT could track BP with quite desired accuracy. Similarly, based on the M-K equation and Bramwell–Hill (B-H) equation [3,12], a novel PTT-BP model by Zheng et al. [17] was developed. During a BP monitoring experiment measuring smooth changes over 24 h, this PTT-BP model was validated as having a good performance regarding BP estimation. However, it was meaningful and necessary to investigate the estimated BP performance in conditions of wild BP fluctuations such as intense exercise and excessive tension or fatigue, instead of the condition of smooth changes in BP.

Recently, heuristic modeling was investigated through regression technique, such as linear estimation [18] and non-linear estimation [3,17,19,20], to estimate BP from the indirect indicators, where the indicators included PTT or heart rate (HR) [21], PIR PPG intensity ratio [14], TDB, a kind of arterial stiffness index denoted the duration from the maximum derivative point to the dicrotic peak in the PPG [21], and others. Furthermore, some reports on predictive modeling with data-driven methods such as machine learning have received more attention to obtain models for better describing the relationship between BP indicators and BP through analyzing pulse wave morphology based on more comprehensive and available indicators from ECG or PPG signals [22,23,24]. Although these BP monitoring solutions described above were helpful, their accuracy of estimating BP still needed to improve to meet the Advancement of Medical Instrumentation (AAMI) standard [25] with a better accuracy.

Referring to the difference of calibration methods during BP estimation models, it could be divided into two categories: the least square method (LS) [5,12,13,26] and point-to-point (PTP) pairing method [3,11,14]. Here, the personalized calibration procedure is employed to obtain unknown parameters of a special BP model before BP monitoring. Once these unknown parameters are determined, they will not change during subsequent long-term BP monitoring. The LS method could not meet the needs of a small initial sample size; for example, some samples were obtained from 5-min signals [11,18] in the initial calibration process due to the requirement of all datasets for long-term BP monitoring. Notably, strictly speaking, the LS method is only suitable for BP estimation in the short time, and it is not suitable BP monitoring for a long time. More importantly, the accuracy of BP estimation depends on the sample/point size for this LS method.

In contrast to the LS method, the PTP paring calibration method (abbreviated as the PTP method or PTP) had become popular in wearable system and cuff-less BP monitoring technology [3,11,14] due to requiring one base value of point/sample to complete the personalized calibration procedure theoretically. However, utilizing different samples/points in the personalized calibration procedure will obtain different unknown parameters of specific models through the function mapping relationship between functions and variable/samples. Therefore, this PTP method is bound to have a huge effect on the initial value sensitivity; to some extent, it reduces and weakens the accuracy of BP estimation. To our knowledge, physiological parameters such as PTT and BP are not always constant in a calm state for subjects and could fluctuate in a range of small variations to be observed. However, for the common one-point PTP (oPTP) pairing calibration method using only one BP paired with one PTT, there was no evidence of improvement so far to explain enough of the entire calm and rest state. Hence, the calibration method needed to be further improved and explained with more detail, especially in the selection of initial samples.

In this study, two calibration methods were researched to weaken the initial value sensitivity of the oPTP method. The first method called the mean point-to-point pairing (mPTP) calibration method was proposed to balance and represent the entire calm and natural breathing state for each subject. Based on the mPTP method, the second calibration called penalty factor point-to-point pairing (fPTP) calibration method was investigated to adjust and correct BP estimation errors in real time. Under three different exercise intensities, the performance of these two calibration methods had been analyzed and compared with the oPTP method through consistency, correlation, and overall performance comparison. Meanwhile, among two most widely studied BP estimation models [3,17], their sensitivities were investigated in the conventional oPTP calibration method and two newly proposed mPTP and fPTP methods, respectively. This study demonstrated a new paradigm to provide a cost-effective cuff-less BP monitoring technology with an easy and durable calibration method.

## 2. Methods

### 2.1. Two Popular Cuff-Less BP Estimate Models

Central arteries pushed blood to narrow distal arteries by the pressure of circulating blood on the walls of blood vessels, thus forming the phenomenon that it expanded during systole and contracts during diastole. Here, the circulatory pressure is blood pressure. Arterial BP, as a hemodynamic parameter, fluctuated on a beat-to-beat basis due to the dynamic interplay from vasomotion, neural regulation, and arterial mechanisms [27].

The fundamental concept of relating BP with PTT is based on the M-K equation [3,12], which modeled a relationship between pulse wave velocity (PWV) and the incremental elastic modulus (a coefficient of elasticity) of the arterial wall or its distensibility [28]. Two popular cuff-less BP monitoring models based on the oPTP calibration method were developed from the M-K equation. One called the MK-BH model [3], introducing the B-H equation [17] into the M-K equation, was employed to overcome the weak linear correlation between PTT and estimating DBP. The other called dMK-BH model [17] established a mathematical relationship between mean blood pressure (MBP) and a factor in which the change in elasticity was caused by pressure wave variations. It could be regarded as the development model of MK-BH. The mathematical relationships between BP and PTT are summarized in Table 1.

Here, *PTT* is the time delay variable for the pressure wave to travel between two arterial sites and obtained from an R peak time interval between the ECG and PPG signal in same cardiac cycle [7,8,9,15]. $PP_{0},\;MBP_{0}\;\mathrm{and}\;PTT_{0}$ are the base values of *PP*, *MBP* and *PTT*, respectively. In the personalized PTP pairing calibration procedure, these parameters’ values will be determined by $PTT_{0}\;$pairing with cuff BP and they will not change during subsequent long-term BP monitoring.

**Figure 1:**
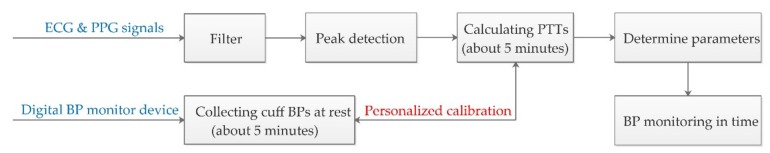
BP monitoring system of including signal process technique.

Figure 1 illustrates the overall architecture of the BP monitoring system. The filter design and experimental protocol are in Section 2.3. After filtering, we detected peaks in both ECG and PPG using Gil et al.’s detection algorithm [29]. Then, PTT was calculated by the time delay between R waves and corresponding peak PPG.

### 2.2. Two Point-to-Point Calibration Methods (mPTP and fPTP) 

The PTT-based BP measurements required an individualized calibration to obtain the unknown coefficients or parameters in the BP estimation model for each subject before monitoring BP. Here, the mapping relationship between BP and PTT is established through paring one *cuff BP* with one PTT, which is called the one point-to-point (oPTP) paring calibration method. However, it was sensitive when using one pair of *cuff BP* and PTT to complete the calibration procedure in the oPTP method. Here, we selected one median of PTT $\left(\hat{PTT}\right)$ from the ECG and PPG signals to pair with one cuff BP for 30 s each for completing one calibration behavior according to the specific BP estimation model.

**Figure 2:**
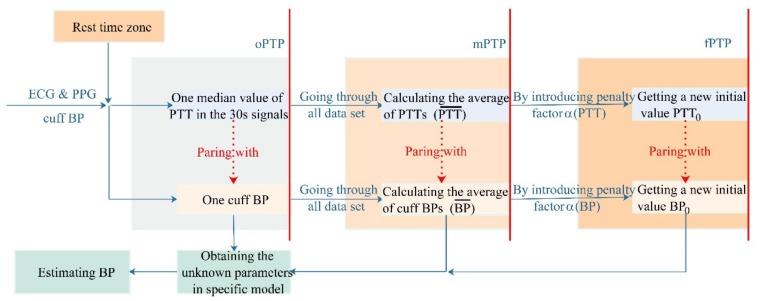
Flowchart of calculation regarding the BP monitoring system in this study.

In the present study, with all the *cuff BPs* and $\hat{PTTs}$ samples during the clam or peace state for about five minutes [3,9,15], we researched two new calibration methods to pair *PTT* with the base value of BP on the basis of the oPTP method. Firstly, a mean point-to-point pairing (mPTP) calibration method was proposed to measure the entire state of calm or rest. Thus, a penalty factor point-to-point (fPTP) pairing calibration method was investigated to adjust and correct the estimated difference in real time. Both the mPTP and fPTP calibration methods overcame the limitation of the initial value sensitivity of the oPTP method, in which BP is taken as a constant in a calm or still state. Their calibration procedures in detail are shown in Figure 2.

To establish the fPTP method, there are two steps: the real-time adjustment of the PTT and the calculation of the base value of the cuff BP.

Step 1: The real-time adjustment of the PTT. It comprises a base value of calibration and a negative feedback function that adjusted and corrected the PTT difference to adapt to the change of cuff BP. Under the condition of calm and natural breathing, their correspondence is shown below.

(1) $$\alpha \left(PTT\right)=\frac{\sum_{i=1}^{n}\left(PTT_{i}-\bar{PTT}\right)}{n\cdot \sum_{i=1}^{n}\left|PTT_{i}-\bar{PTT}\right|}$$

(2) $$PTT_{0}=\bar{PTT}\cdot (1-\alpha \left(PTT\right))$$

where the *PTT_i_* is the *i*-th median of PTT values obtained from the 30 s beat ECG and PPG signals beat by beat. Meanwhile, *n*, *PTT_0_* and $\bar{PTT}$ denoted the sample size, the final calibration initial value of the PTT sequences, and the average value of all PTT samples, respectively. Here, the corresponding PTT feedback function was recorded as $\alpha \left(PTT\right)$ and the based value of calibration is the average PTT ($\bar{PTT}$) obtained in all samples according to the mPTP calibration method.

Step 2: The calculation of the base value of cuff BP. It comprises of a base value of calibration and a negative feedback function responsible for adjusting and correcting the BP estimation errors. Under the condition of calm and natural breathing, their correspondence was shown below.

(3) $$\alpha \left(BP\right)=\frac{\sum_{i=1}^{n}\left(BP_{est_{i}}-BP_{cuff_{i}}\right)}{n\cdot \sum_{i=1}^{n}\left|BP_{est_{i}}-BP_{cuff_{i}}\right|}$$

(4) $$BP_{0}=cuff\bar{BP}\cdot (1-\alpha \left(BP\right))$$

where the *n*, $BP_{cuff_{i}}$ and $BP_{est_{i}}$ denoted the sample size, the *i*-th cuff BP value, and the *i*-th estimated BP value in the special PTT-based BP model whose parameters were obtained by using the mPTP calibration method, respectively. Meanwhile, $BP_{0}$ was the final calibration initial value of the adjusted BP sequences. Here, the corresponding BP feedback function is recorded as $\alpha \left(BP\right)$, and the base value of calibration is the average cuff BP ($cuff\bar{BP}$) obtained in all samples according to the mPTP calibration method.

**Figure 3:**
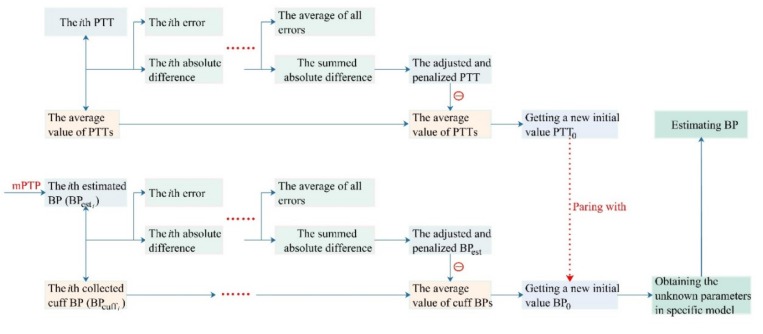
Detailed procedure of the factor point-to-point pairing (fPTP) calibration method.

In summary, the penalty factor $\alpha \left(PTT\right)$ and $\alpha \left(BP\right)$ in the fPTP calibration method consolidated the estimation error of the mPTP method every time and corrected the initial value of monitoring BP in real time. $PTT_{0}\;\mathrm{and}\;BP_{0}$ obtained by traversing all the quiet state samples are sourced as the calibration parameters of the PTT-based BP model. The details about the initial calibration method fPTP with real-time correction and negative feedback adjustment mechanism are shown in Figure 3. 

The calibration was done only once for each subject. After deriving parameters in the BP estimation model, the BP could be estimated continuously.

### 2.3. Experiment and Data Collecting

#### 2.3.1. Experimental Protocol

**Figure 4:**
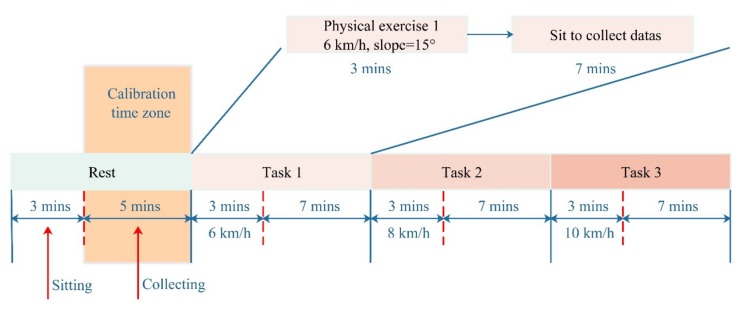
Experimental protocol under different tasks.

This experimental protocol was performed in a study room with temperature 23.9 ± 1.6 °C and Relative Humidity 60–70%. The Powerlab/16sp system (Castle Hill, ADInstruments, Australia, 2002) was employed to synchronously record and amplify the ECG and PPG signals. The ECG signal was filtered by a 1 Hz high-pass filter and a 40 Hz low-pass filter. Meanwhile, the PPG signal was filtered by a 0.5 Hz high-pass filter and a 20 Hz low-pass filter, and the sampling frequency was 1 kHz [15]. The cuff-type BP monitor (HEM-7211-IT, Omron, Japan) was mounted on the right arms of a subject to provide reference BP values. Considering the data sample populations and the replicability of the experimental results, the subjects were required to conduct a resting experiment and exercise a recovery experiment, which took about 35 min in total. The exercise recovery experiment was subdivided into three tasks (i.e., task 1, task 2, and task 3), in which the subjects were told to run on a treadmill (Powereach PET-90, Shanghai, China) with a slope of 15° at 6, 8, and 10 km/h for 3 min, respectively. Then, signals were collected for seven minutes immediately after each end of the running exercise. The details of the experiment are shown in Figure 4. 

#### 2.3.2. Subjects and Data Collecting

A total of 21 subjects without a history of cardiovascular or neurological disorders participated in this study, and the details are listed in Table 2. All the subjects volunteered to participate and gave their written consent before taking part in this study. The study was approved by the Ethnical Committee at the University of Shanghai for Science and Technology.

Each subject was required to complete three running exercises under different protocols for three minutes, which lead to a greater BP change range and guaranteed a more accurate performance in the specific BP model estimation [30]. Once the running exercise was finished, they were asked to sit upright and measure the cuff BP, the ECG, and the PPG signals. Specifically, they were asked to sit on chairs that were 25 cm away from the tables, with the cuff wrapped around their right arm at the same level between the center of the right arm and the subject’s heart. The PPG sensor was placed on their left hand to avoid the effect of cuff inflation on the PPG signals [3]. Additionally, carrying mobile phones and wearing devices were not allowed during the data collection. To avoid signal interference caused by movement, subjects were also required to remain stable and breathe naturally.

### 2.4. Data Analysis

In the present performance of the protocol, more than 100,000 heartbeats were taken and analyzed. Moreover, about 5000 heartbeats were studied for each subject. The estimated BP was calculated based on PTT obtained from a 30 s period of ECG and PPG signals. A total of 1092 pairs of *cuff BP* versus $\hat{PTT}$ (including an individualized calibration process of about 5 min) were found, and 42 pairs of *cuff BP* versus $\hat{PTT}$ during the 21-min recovering experiment were applied while estimating the BP for each subject. The estimated errors between the cuff BP and the estimated BP were evaluated as the mean error (*mean*) ± standard deviations (SD) as well as the mean absolute difference (MAD).

## 3. Results

Two point-to-point calibration methods proposed in this study and one common oPTP calibration method were evaluated and compared regarding their consistency and overall performance in the MK-BH model and dMK-BH models, respectively.

### 3.1. Consistency

**Figure 5:**
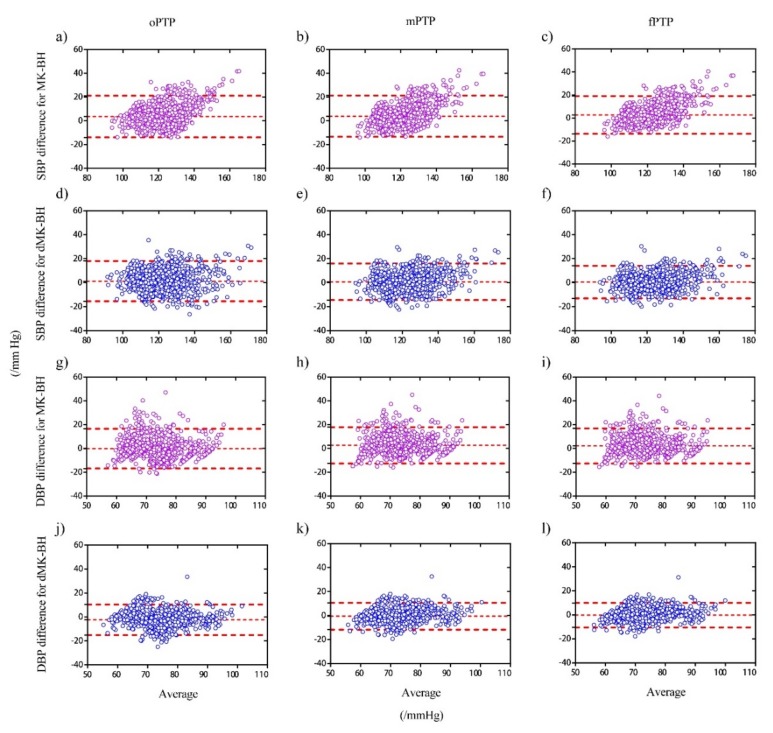
Bland–Altman plots of cuff BP versus estimated BP with one point-to-point (oPTP), mean point-to-point (mPTP), and fPTP method employing for the MK-BH and dMK-BH model in all subjects. Note 1: Three different calibration methods in the MK-BH model for SBP (**a**–**c**) and DBP (**g**–**i**), respectively. Note 2: Three different calibration methods in the dMK-BH model for SBP (**d**–**f**) and DBP (**j**–**l**), respectively. Note 3: The distribution of the difference between the estimated BP and the reference BP was subject to normal distribution.

The Bland–Altman plots was used to analyze the consistency of the BP measures from different BP models and graphically reflect the Limit of Agreement. In Figure 5, both cuff BP and estimated BP with oPTP, mPTP, and fPTP calibration methods were compared in the MK-BH and dMK-BH models for all subjects.

As shown in Figure 5, the estimations agreed well with the references, with 95% of the differences within the agreement area. For the three initial calibration methods, the SBP estimation is more accurate than the DBP estimation in the MK-BH and dMK-BH models. Meanwhile, the fPTP calibration with a penalty factor showed the narrowest 95% Limits of Agreement for the dMK-BH and the MK-BH model. In order to more clearly clarify the performance of each initial calibration method under different models, statistics of Bland–Altman plots are shown in Table 3 and Table 4.

In Table 3, the Bias, Bias of SD, 95% Limits of Agreement, and the within Agreement were collected to report the degree of difference between estimated BPs and cuff BPs in all subjects. For the fPTP method, both the Bias and the Bias of SD were the lowest compared to others for estimating SBP. Specifically, among the dMK-BH for estimating SBP, the Bias and Bias of SD with 0.54 mmHg and 6.95 mmHg is the lowest, and for the MK-BH for estimating SBP, the Bias and Bias of SD with 2.73 mmHg and 8.38 mmHg is the lowest compared to the mPTP method and the conventional oPTP method. The limit of Agreement within ± 1.96 × SD is 94.12% for SBP by using the fPTP method to calibrate the dMK-BH model.

The detailed information about Bland–Altman plots for DBP is shown in Table 4. The predicted DBP by using the mPTP and fPTP methods enhanced the accuracy of estimating BP compared to the conventional calibration, i.e., the oPTP method. Similarly, for estimating SBP, both the Bias and the Bias of SD in the fPTP method are the lowest compared to others for the dMK-BH model. Meanwhile, the Limit of Agreement within ± 1.96 × SD is 93.72% for DBP in the specific dMK-BH model through using the fPTP method. Compared to the common oPTP method, the percentage of fPTP had been increased by 4.05 in the Limit of Agreement for dMK-BH.

### 3.2. Correlation

The correlations between estimated BPs and cuff BPs for MK-BH and dMK-BH models by using different calibration methods were investigated in Table 5.

As shown in Table 5, for the MK-BH and dMK-BH models, the correlation coefficients of DBP estimations are distinctly less than those of SBP estimations by using oPTP, mPTP, and fPTP. Moreover, the dMK-BH model could provide a better correlation with both SBP and DBP than the MK-BH model for each initial calibration method, especially for estimating DBP. Additionally, using the conventional oPTP method, the correlation coefficients for SBP and DBP estimation are the smallest compared to the proposed mPTP and fPTP methods for MK-BH and dMK-BH models. By contrast, using the fPTP method, the correlation coefficients for SBP and DBP estimation are the largest compared to the oPTP and mPTP method for the MK-BH and dMK-BH models. More specifically, for the MK-BH model, using the oPTP method, the correlation coefficients for SBP and DBP estimation are 0.76 and 0.53, respectively. For the dMK-BH model, using the fPTP method, the correlation coefficients for SBP and DBP estimation are 0.87 and 0.78, respectively. 

### 3.3. Overall Performance 

**Figure 6:**
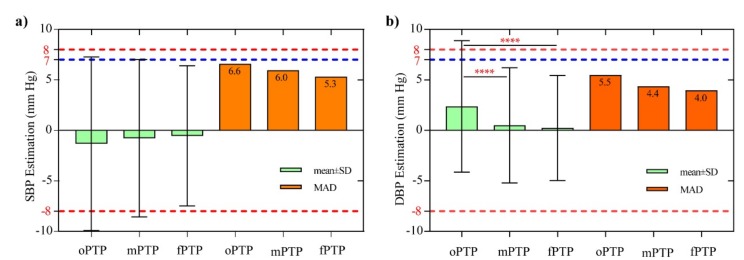
Performance comparison during three different calibration methods in the dMK-BH model for (**a**) SBP and (**b**) DBP. Note 1: The red and blue dotted line denotes the largest boundary for the SD (8 mmHg) and mean absolute difference (MAD) (7 mmHg). Note 2: Significant differences: *p < 0.05, **p< 0.01, ***p< 0.001, and ****p< 0.0001.

As mentioned earlier, the BP estimation performance of the dMK-BH model is superior to the MK-BH model. Hence, the performance of the dMK-BH model by the means of three different initial methods is further analyzed as shown below. Another criterion for the performance evaluation includes mean ± SD, MAD, and SD of estimations for the MK-BH model by the means of oPTP, mPTP, and fPTP. Differences were tested using one-way ANOVA with multiple comparisons via the Turkey test to determine whether statistically significant differences were observed among the oPTP, mPTP, and fPTP methods. These comparisons are further shown in Figure 6.

Referring to Figure 6, the oPTP method shows the largest mean ± SD (MAD) of -1.326 ± 8.576 (6.6) mmHg for estimating SBP error and 2.377 ± 6.512 (5.5) mmHg for estimating DBP error, respectively. Clearly, both the mPTP and fPTP methods weaken the estimated BP errors. For instance, the SBP estimation error of the mPTP and the fPTP is decreased by 0.6 and 1.3 mmHg regarding the MAD, respectively. Similarly, the DBP estimation error of the mPTP and the fPTP is decreased by 1.1 and 1.5 mmHg regarding the MAD, respectively. It is noteworthy to mention that the mean errors and the SD of the errors is within 5 and 8 mmHg for estimating BP for the dMK-BH model by using the mPTP and fPTP methods. Consequently, it is consistent with the AAMI requirements of 5 ± 8 mmHg (mean ± SD) for a BP estimation error [25]. However, the SD (8.58 mmHg) of the errors obtained by the oPTP method slightly did not meet the requirements of AAMI based on our experiments. Notably, there was a statistically significant difference between the traditional oPTP method and the mPTP method for estimating BP based on PTT (*p* value < 0.0001). Similarly, a statistically significant difference between the oPTP method and proposed fPTP method was also observed (*p* value < 0.0001). Additionally, there was no statistically significant difference found between oPTP and mPTP/fPTP.

**Figure 7:**
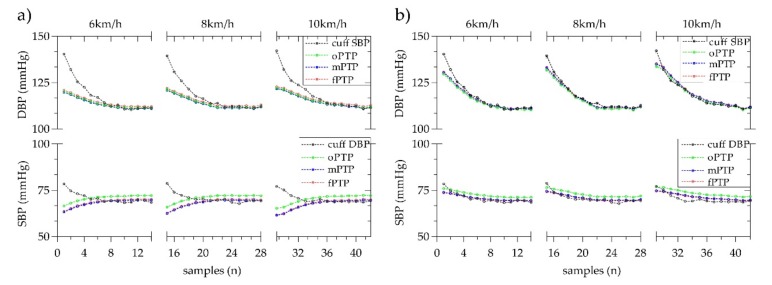
The mean change trend of estimating BP using the oPTP, mPTP, and fPTP methods under three exercise intensities for (**a**) the MK-BH model and (**b**) the dMK-BH model for all subjects.

Figure 7 shows the mean estimated BP trend under different exercise intensities, i.e., running exercise in 6, 8 and 10 km/h. The estimation of both MK-BH and dMK-BH models are superior to the estimation using the conventional oPTP method, especially for estimating DBP when the mPTP and fPTP are selected as the initial calibration methods to estimate BP. Besides, for each initial calibration method, the performance of the MK-BH model is lower than that of the dMK-BH model with its different exercise intensity. Compared to the oPTP method, the mPTP and fPTP methods could be used to improve a better BP reading with 3–5 mmHg and make the estimated BP closer to the cuff BP. Moreover, the estimated BP from fPTP method is further closer to the cuff BP than the mPTP method. It is worth mentioning that the change trend of the estimated DBP and cuff DBP is completely opposite with the increase of DBP fluctuations by using the MK-BH model.

### 3.4. Comparison with Prior Works

Our study achieved comparable results to the rest of the studies. Here, all results are presented in both mean ± SD (MAD) and Pearson correlation coefficient (i.e., r). Table 6 presents a comparison of the results reported in this paper with the results reported in the literature.

According to Table 6, considering the published results, an error of ±10.02 mmHg for SBP and ±10.03 mmHg for DBP is achieved in a pilot study only using an ECG signal with 51 subjects [31]; the achieved error for SBP and DBP estimation is 2.16 ± 6.23 (8) mmHg and −1.49 ± 6.51 (9) mmHg, respectively, in a case study only using a PPG signal with 26 subjects and matching the SBP and DBP values [32]; −0.03 ± 8.58 (9) mmHg for SBP and −0.02 ± 5.81 (7) mmHg for DBP is obtained from a method that uses ballistocardiography (BCG) and PPG signals with an intervention of deep breathing and sustained handgrip for 51 subjects [33]. Notably, in Kim et al.’s investigation [33], the correlation coefficient is 0.70 and 0.60 for SBP and DBP, respectively. Similarly, 0.31 ± 8.55 (5) mmHg for SBP and −0.5 ± 5.07 (4) mmHg for DBP is obtained from a method that uses impedance plethysmography (IPG) and PPG signals with 51 subjects; also, the correlation coefficient is 0.88 for BP [34]. Additionally, some multi-wavelength and machine learning methods were also investigated. For instance, an estimated error is reported of 0.00 ± 2.85 (2.2) mmHg for SBP and 0.00 ± 1.75 (1.4) mmHg for DBP by using ECG and PPG signals with an intervention of deep breathing and a Valsalva maneuver from a multi-wavelength method [36]; and a similar machine learning method tested on 772 datasets from the MIMIC-III database [37] for approximately 155 subjects provides an error of 0.03 ± 5.52 (3.27) mmHg for SBP and 0.10 ± 1.97 (1.16) mmHg for DBP. In addition, the correlation coefficient is 0.97 for BP [22].

## 4. Discussion

A key challenge in using the PTT-based BP measurements is required by an individualized calibration. However, the conventional oPTP calibration method [3,5,12,13,26] is unreliable to offer full insights into BP variations in different situations because it only used one *PTT* paring with one *cuff BP* to obtain unknown parameters in a specific BP estimation model. Consequently, this oPTP method has initial sensitivity during cuff-less BP monitoring. It is expected to improve the oPTP method by the introduction of some extra parameters or datasets that were indicative of BP changes in the initial calibration process.

In this study, we researched two new mPTP and fPTP methods to better estimate BP and compared them with the oPTP method in the MK-BH and dMK-BH models. Firstly, we proposed the mPTP method with averaging all datasets at rest. It is a mean estimation in the median value of PTTs obtained from ECG and PPG signals in whole calm or peace state. Compared to the traditional oPTP method, the mPTP method has a better performance for BP estimation as exhibited in Section 3. Additionally, the fPTP method was introduced to adaptively correct the estimated error based on the mPTP method as presented in Section 2.2. For the fPTP method, the penalty factor consolidated the estimation error of the mPTP method every time and corrected the initial value of monitoring BP in real time. The results obtained from the experimental protocol have clearly indicated, for this fPTP method, that the MK-BH or dMK-BH models have the largest correlation coefficient, the smallest consistency interval, and the lowest estimated errors between the estimated BP and cuff BP compared to others (see Section 3). Here, note that cuff BPs and estimated BPs are skewed variables. In other words, it is unrealistic and incorrect to distinguish between dependent variables and independent variables according to correlation in both cuff BPs and estimated BPs. More importantly, both the mPTP and fPTP pairing calibration methods are easy to implement, and only a five-minute samples at rest is required to complete the specific personalized calibration procedure. Furthermore, their individualized calibration was executed only once at the beginning of the BP monitoring.

Compared with the oPTP method, our proposed mPTP and fPTP methods showed a statistically significant difference during estimating DBP (see Figure 6). It is important information that our study achieved comparable results compared to the rest of the studies [22,31,32,33,34,35,36] (see Table 6). To be more specific, the error for SBP and DBP estimation is −0.77 ± 7.79 (6.0) and 0.51 ± 5.70 (4.4) mmHg; and the correlation coefficient is 0.83 and 0.74 for SBP and DBP, respectively, using the mPTP method. Similarly, the achieved error for SBP and DBP estimation is −0.54 ± 6.95 (5.3) and 0.24 ± 5.21 (4.0) mmHg; and the correlation coefficient is 0.87 and 0.78 for SBP and DBP, respectively, in using the fPTP method. Other information worth mentioning is that the error between the estimated BP and the cuff BP decreases as the BP increases due to the increased exercise intensity for healthy subjects (see Figure 7). This indicates that the MK-BH and dMK-BH models are more suitable for estimating large fluctuations in BP than fluctuations with a small range and that the initial calibration methods proposed in this study are more practical, especially in estimating DBP. This fact is valuable, since in many studies [3,9,10,12,38,39,40], compared to DBP, the estimation errors associated with SBP were considerably less. 

No matter which initial calibration method is used, the dMK-BH model is superior to MK-BH in BP estimation (see Table 3, Table 4 and Table 5, Figure 5, Figure 6 and Figure 7). The basis of the modeling sources indicated the variability of BP estimation performance. The M-K equation had provided a mathematical foundation for advanced research toward the direction of non-invasive BP monitoring. To strengthen the correlation between the estimated DBP and cuff DBP, Tang et al. [3] obtained the MK-BH model by introducing the Bramwell–Hill (B-H) equation into the M-K equation. Furthermore, based on the MK-BH model, Poon et al. [17,41] introduced MBP into the MK-BH model to better estimate BP and developed a new cuff-less BP estimation model called the dMK-BH model. Hence, the introduction of MBP to the dMK-BH model is the main reason for its greater accuracy for BP estimation than the MK-BH model. This reveals that MBP is a key factor in the cuff-less BP estimation model. Here, it is necessary to point out that there are some limitations should be further investigated in estimating DBP for the MK-BH and dMK-BH models. More specifically, when estimating DBP, the MK-BH model comprises of two parts: a linear function with SBP and a power function without SBP. This suggests that the DBP might decrease with an increase of SBP, which is inconsistent with the situation in which the SBP varied with the same trend as DBP during experiment. For the dMK-BH model, γ as a physiological parameter is reported to change with aging [17,18,32] and the development of cardiovascular diseases [42]. It is not easy to obtain an optimal γ value in different ages and pathophysiologic conditions.

Recently, improvements of the initial calibration accuracy for cuff-less BP estimation models were investigated. For instance, the covariates such as HR [40], PWV [43], and PIR [12,27] were also introduced into calibration methods and estimation models to better predict BPs. However, with the introduction of these covariates, the amount of computation will increase sharply. Of equal importance to initial calibration, some investigations mentioned that periodic calibration should be considered to improve the reliability of BP measurement since the period between calibrations is short and possibly affected the accuracy of parameters [44]. Our fPTP method with adjusting and penalizing behavior proposed in this study provided an effective and practical way to solve the periodic calibration by introducing it into a specific model.

One limitation of this study is that the subjects are generally young and healthy. A larger cohort study including individuals recruited from wide age ranges and various or some specific cardiovascular diseases will be conducted to further validate the proposed calibration methods in the revised estimated BP model for ABPM and HBPM. Meanwhile, more in-depth measurements including ECG [31], PPG [3,8,9,11,45], BCG [33], IPG [34], ICG [36], and others [22,31,36] need to be involved in future research to fully verify this work. Additionally, the M-K equation, as a mathematical foundation for advancing research toward the direction of non-invasive BP monitoring, implies several assumptions [3,17], which might be invalid for complex behavior and for regulation of the involved arterial tree, such as the thickness-to-radius ratio [39,46], which is seen as a constant.

## 5. Conclusions

In this study, we proposed two new point-to-point calibration methods, that is, mPTP with averaging peace or rest status and fPTP with a penalty factor to automatically correct errors. Together with the conventional oPTP method, their respective BP estimation performances are compared in two popular non-invasive PTT-based BP models. Compared to the oPTP method, the mPTP method could provide a stronger correlation and greater consistency with cuff BP and estimated BP. Moreover, the lowest errors were found in the fPTP calibration method than others. Therefore, the fPTP method for calibration in estimating BP could be employed for the accurate estimation of both SBP and DBP targets in the BP estimation model based on PTT index (obtained from PCG and PPG signals). The calibration method with plenty of factors in this study was explored for the first time and it may blaze a new paradigm in physiological monitoring and assessment with a periodic calibration for a better PTT-based BP reading.

## Figures and Tables

**Table 1:** Two commonly estimating blood pressure (BP) models. SBP: systolic blood pressure, DBP: diastolic blood pressure, MK-BH: combination of the Bramwell–Hill and Moens–Korteweg equations, dMK-BH: development model of MK-BH.

Models	SBP	DBP
MK-BH [3]	$SBP_{0}-\frac{2}{\gamma \cdot PTT_{0}}\cdot \left(PTT-PTT_{0}\right)-\frac{2}{{\gamma *\mathrm{PAT}}_{0}}*\left(\mathrm{PAT}-{\mathrm{PAT}}_{0}\right)$	$SBP-PP_{0}\cdot {\left(\frac{PTT_{0}}{PTT}\right)}^{2}$
dMK-BH [17]	$DBP+PP_{0}\cdot {\left(\frac{PTT_{0}}{PTT}\right)}^{2}$	$MBP_{0}+\frac{2}{\gamma }ln\left(\frac{PTT_{0}}{PTT}\right)-\frac{PP_{0}}{3}\cdot {\left(\frac{PTT_{0}}{PTT}\right)}^{2}$

Note 1: γ denotes a vascular information parameter. Note 2: $PP_{0}=SBP_{0}-DBP_{0},MBP_{0}=(PP_{0}+DBP_{0})/3$ , PP—pulse pressure, MBP—mean BP. Note 3:$\;SBP_{0},DBP_{0},\;PTT_{0},\;{\mathrm{SBP}}_{0},{\mathrm{DBP}}_{0},{\mathrm{PP}}_{0}$ are determined at the beginning of monitoring by calibration using an additional cuff-type BP monitor device.

**Table 2:** Characteristics of the subjects. BMI: body mass index.

Selection Factor	Values
Total number (M, F)	21 (14, 7)
Age (years)	22.48 ± 1.03
Height (cm)	171.52 ± 8.14
Body mass (kg)	63.81 ± 11.68
BMI (kg/m^2^)	21.46 ± 2.89
SBP (mmHg)	122.84 ± 13.04
DBP (mmHg)	74.42 ± 6.65

**Table 3:** The details of Bland–Altman plots on SBP estimation.

Methods	MK-BH; dMK-BH/mmHg			
	Bias	Bias of SD	95% Limits of Agreement	within Agreement/%
oPTP	3.56; 1.33	8.95; 8.58	(−13.98, 21.11); (−15.48, 18.13)	86.73; 93.01
mPTP	3.89; 0.77	8.86; 7.79	(−13.47, 21.25); (−14.50, 16.03)	82.07; 93.52
fPTP	2.73; 0.54	8.38; 6.95	(−13.71, 19.14); (−13.08, 14.15)	83.89; 94.12

**Table 4:** The details of Bland–Altman plots on DBP estimation.

Methods	MK-BH; dMK-BH/mmHg			
	Bias	Bias of SD	95% Limits of Agreement	Within Agreement/%
oPTP	−0.14; −2.38	8.53; 6.51	(−16.85, 16.57); (−15.14, 10.39)	85.01; 89.67
mPTP	2.61; −0.51	7.79; 5.70	(−12.65, 17.87); (−11.69, −11.69)	81.46; 93.11
fPTP	2.16; −0.24	7.52; 5.21	(−12.58, 16.91); (−11.69; −11.69)	82.78; 93.72

**Table 5:** The Pearson correlation coefficient (r) between estimated BP and cuff BP.

Methods	SBP		DBP	
	MK-BH	dMK-BH	MK-BH	dMK-BH
oPTP	0.76	0.80	0.53	0.70
mPTP	0.78	0.83	0.54	0.74
fPTP	0.81	0.87	0.56	0.78

**Table 6:** Comparison results with prior work. BCG: ballistocardiography, ECG: electrocardiogram, IPG: impedance plethysmography, PPG: non-invasive photoplethysmography.

Calibration		Subjects	Acquired Signals (Measure Location)	Accuracy w.r.t. cuff BP [*mean* ± SD (MAD)/mmHg; *r*]
Method	Intervention			
Probability distributions [31]	Daily activities	N = 51	Only ECG: chest	SBP:/± 10.22 (7.72);/ DBP:/± 10.03 (9.45);/
oPTP [32]	Supine position	N = 26	Only PPG: ear, toe	SBP: 2.16 ± 6.23 (8^†^);/ DBP: −1.49 ± 6.51 (9^†^);/
oPTP [33]	Deep breathing + Sustained handgrip	N = 15	ECG: chest BCG: foots	SBP: −0.03 ± 8.58 (9) ^†^; 0.70 DBP: −0.02 ± 5.81 (7) ^†^; 0.66
oPTP [34]	handgrip exercises	N = 15	IPG: wrist PPG: finger	SBP: 0.31 ± 8.55 (5^†^); 0.88 DBP: −0.5 ± 5.07 (4^†^); 0.88
PTP, three pairs [35]	Post-exercise	N = 10	PPG: finger Bio-Z: wrist	SBP: −0.09 ± 7.16 (6^†^); 0.86 DBP: −0.02 ± 2.56 (5^†^); 0.77
Multi-wavelength [36]	Deep breathing + Valsalva maneuver	N = 40	ECG: wrist, foot PPG: finger ICG: neck, thorax	SBP: 0.00^†^ ± 2.85 (2.2); 0.98 DBP: 0.00^†^ ± 1.75 (1.4); 0.99
Machine learning [22]	MIMIC-III [37]	N = 155^†^	ECG and PPG [37]	SBP: 0.03 ± 5.52 (3.27); 0.97 DBP: 0.10 ± 1.97 (1.16); 0.97
mPTP, this work (dMK-BH model)	Running exercise	N = 21	ECG: wrist, foot PPG: finger	SBP: −0.77 ± 7.79 (6.0); 0.83 DBP: 0.51 ± 5.70 (4.4); 0.74
fPTP, this work (dMK-BH model)	Running exercise	N = 21	ECG: wrist, foot PPG: finger	SBP: −0.54 ± 6.95 (5.3); 0.87 DBP: 0.24 ± 5.21 (4.0); 0.78

Note 1: “/” Not be estimated based on reported results or able to be reported in corresponding authors’ other work. Note 2: “^†^” Be approximately estimated from the corresponding Bland–Altman plots.
